# A Sunday Lecturer

**Published:** 1890-03-22

**Authors:** 


### The Doctor's Pulpit.

A SUNDAY LECTURER.

Sir Henry Roscoe, M.P., D.C.L., F.R.S., may be taken as a fairly typical representative of the "men of science" of the present time. The Sunday Lecture Society may be looked upon as the popular church—or shall we say the mission hall?—of scientific enthusiasts. A man who might be desirous of knowing something about the leading thought currents of his time would not unnaturally go to hear the Sunday lecturers. He would expect therefrom to be able to form a judgment on the relative practical potency of scientific men as compared with other intellectual leaders of our own era. For the mass of mankind are still led, as in all former ages, by a few spirits that are bolder, more energetic, and more self-asserting than their fellows. Not only are such leading spirits well worthy of consideration in themselves, but he who wishes to have an intelligent understanding of his own times, either for practical or for philosophic purposes, must bend the best energies of his mind upon them, or remain in hopeless mental chaos so far as the immediate present is concerned. In such a temper of mind as is here indicated the writer accepted a special invitation to hear Sir Henry Roscoe deliver a Sunday lecture on "M. Pasteur and his Work."

Sir Henry is a broad, stout, bluff, fox-hunting-looking man, apparently about sixty years of age. He gives the impression of being well fed, prosperous, and of an easy turn of mind; by no means the sort of person to set the Thames on fire either in the cause of science, or any other cause. He speaks quietly, deliberately, and sometimes with a little hesitancy—a kind of half stammer. On Sunday week, as has been said, he was to discourse about Pasteur and his work; and as he may be regarded almost as Pasteur's *alter ego* in this country, a good deal was expected of him by the writer, and no doubt by many other persons. St. George's Hall, in which the lecture took place, was well filled by a very attentive and expectant audience.

A born speaker soon shows his mettle and his quality. Sir Henry Roscoe is no speaker. A vivid recollection of the literary deficiencies of "Roscoe's Chemistry" had prepared the writer to expect that. The lecture was to last an hour. The first quarter of this limited period was spent in the delivery of a series of quite uninteresting and useless generalities: the matter was, if truth must be spoken, mere padding. Not only was it padding, but it was poor, aimless, commonplace, and excessively stale padding. Even Exeter Hall in the palmy days of its well-remembered lectures could hardly have matched it. Those who had gone to St. George's Hall expecting a first-hand exposition and vindication of Pasteur's treatment for hydrophobia were very impatient at the lecturer's delay in coming to the real substance of his argument. According to the syllabus which had been given to visitors as they entered the hall, there were many points to be discussed before the hydrophobia question was reached. That, indeed, had been reserved for the peroration; and it became clear that unless the speaker should quicken his pace the most burning, if not the most important, part of his subject would fail to be reached. From a medical man's point of view, and one anxious to hear, see, and comprehend Pasteurism from Pasteur's own standpoint, this slow progress was more than disappointing. For there is no doubt that to medical men, at any rate, the extensive series of questions raised by Pasteur's inoculations for hydrophobia are of more consequence than all his other discoveries put together. To discuss Pasteur and his work without considering in detail his anti-rabic treatment is like acting the play of Hamlet with the Prince of Denmark left out.

When the lecturer came at length to his subject, it was, to say the least, depressing to see the way in which he handled it. There is, perhaps, nothing very poetical or inspiring in the phenomena of fermentation; and no doubt Sir Henry Roscoe has so often discoursed to junior chemistry students on the yeast plant and its rapid multiplication, and the remarkable effects produced by its growth upon the contents of a beer vat, that he probably found it difficult to get up any enthusiasm. But surely something more is expected of a Sunday lecturer and an eminent scientist, when he addresses a popular audience, than that he should play the part of a mere perambulating primer of science. It is true he showed microscopic preparations of torulæ and other allied organisms on a screen by means of the oxy-hydrogen light, and displayed considerable familiarity with the use of the long pointer. But most of this can be found in the first chapters of any text book of elementary physiology. What was expected of Sir Henry Roscoe was not a class-room demonstration, but a philosophical exposition of the facts and principles of the science of minute organisms, of their relation to one another and to their hosts, and of the possibility of controlling any injurious tendencies they may have by rational and efficient measures. The audience had come out, in short, to see an earnest and, perhaps, heroic pioneer of science wrestling with the mysteries of organic life in its most mysterious forms. Poor Sir Henry Roscoe! He gave the impression of having had too large a piece of indigestible pudding for lunch much more than of being in deadly earnest, grappling with problems of life and death. Perhaps he thinks it bad form to be too much in earnest, though considering the side of the House of Commons on which he sits we should hardly expect that.

Pasteur's discoveries in connection with the silk-worm disease, chicken cholera, and anthrax, were passed in rapid review. But here again the "primer" style of oratory was adopted. It seemed to be taken for granted by the speaker that the audience knew little or nothing about the facts he was discussing, and neither knew nor cared anything at all about the philosophy of the facts. Now it is a very good rule for a speaker to take it for granted that his audience will be grateful for a clear statement of facts. But it is a very bad rule for any speaker to assume that the most ignorant of audiences cares nothing about principles and arguments. It must never be forgotten that man, by his very nature, is a reasoning animal, and that the most important fact in the world is comparatively uninteresting to him until it is illuminated by its underlying philosophy and reason. Of this simple generalization Sir Henry Roscoe seems to be profoundly ignorant. The effect produced on the minds of some scientific hearers, with regard to anthrax and chicken cholera, was that it was quite impossible to determine whether Pasteur had or had not made any really valuable discoveries; and that Sir Henry Roscoe had no guiding hypotheses to offer whereby trained minds might construct a doctrine of probabilities or of improbabilities for themselves.

Before the statement of facts regarding anthrax and chicken cholera was finished the hour was on the stroke of five. The Pasteurists and the anti-Pasteurists felt equally that they were going to be disappointed. Unless the lecturer asked for more time he could not even state the case for anti-rabic inoculation, much less illustrate and prove it. He did, however, set out a statement, and took it for granted that since he and other scientists were satisfied of its value nothing more needed to be said. But what kind of a position is this for science to take up? Exactly the position assumed by the Church of Rome, with her infallible head, on questions theological and ecclesiastical. It has come to this, then, that if one eminent scientist arrives at a definite conclusion and a few other eminent scientists agree with him, the world is to take the conclusion solely on their authority. But the world will not do that, and ought not to do it. The world demands to have its reason convinced and its intelligence satisfied. There is no such thing in science as a fact or a principle which cannot be comprehended by a non-scientific man of competent intelligence, provided the non-scientific man chooses to devote sufficient time and labour to the question. If, as seems imminent, we are to be trampled upon by an infallible scientific tyranny it will be necessary to draw up a "Magna Charta," securing the rights of the "plain man." One of the first principles of such a charter must be the affirmation of the right of the plain man to have his intelligence satisfied by every scientific dogma he is asked to accept. There is a sense in which scientific salvation is by faith; but the faith, to be of any value, must be based on the conviction that what is believed can at any convenient time be clearly demonstrated to every mind willing to give adequate attention to the demonstration.

It is saying little to affirm that Sir Henry Roscoe has not proved the scientific value of Pasteur's anti-rabic treatment. He did not attempt to prove it in his address. So far from proving it he did not even seem to see or understand the obvious difficulties of the case. He did indeed admit it to be a peculiar circumstance that the inoculation of an animal with a weak virus, after the animal had been previously inoculated with a stronger virus of the same kind, should prevent the natural development of the earlier and more potent poison. But he did not offer any explanation of this remarkable and unparalleled phenomenon. The still more striking circumstance that, contrary to all analogy, the weak virus, which ought at least to produce in the inoculated a modified hydrophobia, produces no symptoms whatever, was not so much as mentioned. Not only was there no attempt at showing any kind of scientific rationale in what has come to be known as Pasteurism, there was not even a presentment of statistical arguments. A few general statements were made, the "sentimentalists" were mildly denounced, the onward march of science, in spite of wicked opponents, was confidently predicted, a very good portrait of Pasteur was thrown upon the screen in the full blaze of the oxy-hydrogen light, and then a most unsatisfactory "Sunday lecture" was brought to a close. The Sunday Lecture Society must do better than this if it has any anticipation of future laurels.

